# Independent predictors for 90-day readmission of emergency department patients admitted with sepsis: a prospective cohort study

**DOI:** 10.1186/s12879-021-06007-9

**Published:** 2021-04-01

**Authors:** Peer Oscar Overgaard Stenholt, S. M. Osama Bin Abdullah, Rune Husås Sørensen, Finn Erland Nielsen

**Affiliations:** 1 Department of Emergency Medicine, Copenhagen University Hospital, Bispebjerg and Frederiksberg, Ebba Lunds Vej 40A, Entrance 67, 2400 NV Copenhagen, Denmark; 2 Department of Emergency Medicine, Slagelse Hospital, Slagelse, Denmark; 3 Department of Anaesthesiology and Intensive Care, Odense University Hospital, Odense, Denmark; 4 Copenhagen Center for Translational Research, Copenhagen University Hospital, Bispebjerg and Frederiksberg, Copenhagen, Denmark

**Keywords:** Emergency department, Sepsis, Readmission, Predictors

## Abstract

**Background:**

The primary objective of our study was to examine predictors for readmission in a prospective cohort of sepsis patients admitted to an emergency department (ED) and identified by the new Sepsis-3 criteria.

**Method:**

A single-center observational population-based cohort study among all adult (≥18 years) patients with sepsis admitted to the emergency department of Slagelse Hospital during 1.10.2017–31.03.2018. Sepsis was defined as an increase in the sequential organ failure assessment (SOFA) score of ≥2. The primary outcome was 90-day readmission. We followed patients from the date of discharge from the index admission until the end of the follow-up period or until the time of readmission to hospital, emigration or death, whichever came first. We used competing-risks regression to estimate adjusted subhazard ratios (aSHRs) with 95% confidence intervals (CI) for covariates in the regression models.

**Results:**

A total of 2110 patients were admitted with infections, whereas 714 (33.8%) suffered sepsis. A total of 52 patients had died during admission and were excluded leaving 662 patients (44.1% female) with a median age of 74.8 (interquartile range: 66.0–84.2) years for further analysis. A total of 237 (35,8%; 95% CI 32.1–39.6) patients were readmitted within 90 days, and 54(8.2%) had died after discharge without being readmitted. We found that a history of malignant disease (aSHR 1,61; 1.16–2.23), if previously admitted with sepsis within 1 year before the index admission (aSHR; 1.41; 1.08–1.84), and treatment with diuretics (aSHR 1.51; 1.17–1.94) were independent predictors for readmission. aSHR (1.49, 1.13–1.96) for diuretic treatment was almost unchanged after exclusion of patients with heart failure, while aSHR (1.47, 0.96–2.25) for malignant disease was slightly attenuated after exclusion of patients with metastatic tumors.

**Conclusions:**

More than one third of patients admitted with sepsis, and discharged alive, were readmitted within 90 days. A history of malignant disease, if previously admitted with sepsis, and diuretic treatment were independent predictors for 90-day readmission.

## Background

Sepsis is a common and serious condition with high mortality. It has been estimated that more than 30 million people are affected each year, and with more than 5 million deaths annually worldwide [1].

We do not have a robust definition of sepsis. Since 1992 sepsis has been defined as a clinical syndrome that required at least two Systemic Inflammatory Response Syndrome (SIRS) criteria in the presence of suspected or confirmed infection [2, 3]. However, since SIRS lacks sensitivity and is not specific, new criteria for sepsis (Sepsis-3) was introduced in 2016 [4]. Sepsis is now defined as a life-threatening organ dysfunction due to a dysregulated host response to infection. Organ dysfunction is defined as an increase of two points or more from baseline in the Sequential Organ Failure Assessment (SOFA) score [4]. A simplified version of SOFA, quickSOFA (qSOFA) was introduced to early identification of patients with infections who are at high risk of poor outcome [4] but has been criticized due to a low sensitivity and poor prognostic accuracy [5].

Sepsis can have severe consequences after discharge with physical, cognitive and psychiatric impairment [6] and increased risk of readmission [7]. Readmission is a substantial burden of the health care system and for the patients [7, 8]. However, the literature on readmission after sepsis, and especially after the introduction of the new sepsis-3 criteria, is sparse. The primary objective of our study was therefore to examine the rate of 90-day readmission and predictors for readmission in a prospective cohort of septic patients admitted to an emergency department (ED) who were identified by the SOFA score.

## Methods

### Design and setting

This is a sub-study of the prospective cohort study of the prognostic ability of qSOFA among all adult (≥ 18 years) patients with infectious diseases admitted to the department of emergency medicine, Slagelse Hospital, during the period October 1st 2017 to March 31st 2018 [5]. Slagelse Hospital is a tertiary care center and the ED has an uptake area of 198,000 adult inhabitants with 26,500 visits annually. The Danish health care system offers equal access for all residents. Danish patients who are hospitalized acutely are admitted through the ED of a public hospital. The patients are either referred by general practitioners, arrive directly through the emergency ambulance service or walk in without any preceding contact. The privately funded hospitals in Denmark have no acute patient intake [9].

### Definitions

Patients with infection were defined as those treated with antibiotics (AB) within 24 h from admission to the ED and continued the AB treatment for at least 48 h. Patients with sepsis were defined as patients with an infection and with an increase in the SOFA score of at least two on admission compared to the baseline value. Calculation of the SOFA scores were done retrospectively. Patients without chronic diseases had a baseline SOFA score of zero. We have adjusted the baseline SOFA score for chronic diseases that could have an impact on the SOFA calculation. Patients with chronic diseases (respiratory, kidney, liver) were registered according to the Charlson Comorbidity Index (CCI) [10] and the baseline SOFA score assigned a value from one to four depending on the severity of the chronic disease. This assessment was based on a combination of information on the level of chronicity (mild, moderate or severe kidney and liver disease) from the CCI classification and the arrival creatinine and bilirubin values. The adjustment for chronic pulmonary disease was based on information on pulmonary disease according to the CCI classification and if different grades of decreased arrival PaO_2_ values at the ED were deemed to be chronically reduced. Patients with known dementia was assigned a baseline sofa score of 1. The adjustments of the SOFA scores were based on consensus of three authors (OBA, RHS, FEN).

The variables and definitions included in the qSOFA score [4] and SIRS criteria [2, 3], used in this study were in accordance with the original guidelines.

The CCI score was divided into three levels: low (CCI score 0), moderate (CCI score 1 or 2), and high (score ≥ 3).

New onset atrial fibrillation was defined as episodes of atrial fibrillation documented on a 12-lead electrocardiogram (ECG) on admission and without a history of prior AF.

### Inclusion and exclusion criteria

All patients with infectious diseases and treated with AB at arrival or within 24 h from arrival to the ED were included in the original study [5]. Exclusion criteria were: Foreign nationality without a Danish Civil Registration number, if antibiotic treatment was discontinued within 48 h, prophylactic antibiotic treatment in relation to surgery, registration errors, transfer to another hospital within 24 h, and if previously included during the study period [5].

### Patients with infections

All patients were triaged at the ED arrival by nurses and by use of a standardized electronic triage form. The triage form included main subjective complaints and vital sign parameters: Blood oxygen saturation (%), respiratory rate (RR) (min^− 1^), blood pressure (mmHg), heart rate (HR) (min^− 1^), body temperature (Tp) (°Celsius), and level of consciousness on a Glasgow Coma Scale (GCS) or by use of the Alert-Verbal-Pain-Unresponsive (AVPU) scale. Patients with documented or suspicion of infections fulfilling either qSOFA or SIRS criteria indicative of sepsis on admission to the ED, or if sepsis was suspected despite a qSOFA score < 2 or SIRS criteria < 2, were given priority to rapid examination for sepsis by a physician. The SOFA score was not used as a routine sepsis screening tool during the study period. Septic patients were examined and treated in accordance with a standardized sepsis protocol with oxygen, intravenous fluids, intravenous AB (either piperacillin-tazobactam or ampicillin-gentamicin or in case of allergies cefuroxime-gentamicin or meropenem), ECG analysis, arterial and venous blood sample analysis (hematologic components, organ markers, C-reactive protein (CRP), electrolytes), blood cultures, and identification of the source of infection. After the initial treatment in the ED and if the patients required hospitalization for more than 48 h, they were transferred to a medical ward (department of endocrinology or department of lung diseases). Patients who did not respond to the initial ED treatment and deteriorated were transferred to the intensive care unit (ICU).

### Data collection process

All electronic records and triage forms of adult patients admitted to the ED during the study period were examined by the authors (OBA, RHS) on the following working-day after the index admission for an infection. We obtained the following demographic data, information on triage variables on admission to the ED, admission laboratory tests, results of other examinations (eg, X-ray, ultrasound, CT and gynaecological examinations) and information on medical history: Age, gender, time of arrival to the ED, GCS or AVPU, systolic blood pressure (SBP), RR, HR, Tp, leucocyte count (× 10^9^/L), peripheral oxygen saturation (%), C-reactive protein (CRP), creatinine (μmol/L), bilirubin (μmol/L), platelets count (× 10^9^/L), lactate (mmol/L), glucose (mmol/L), results of blood cultures obtained on admission, ECG rhythm on admission, source of infection, transfer to ICU, treatment in ICU, information on comorbidities (CCI) and medical treatment before admission and time of discharge from the hospital. The recorded data were regularly entered into an electronic database and randomly controlled by the authors [5].

Information on medical history, vital parameters, test results during admission, treatment, and transfer to medical ward or ICU were obtained from the triage forms and electronic patient records.

Information on sources of infections was based on a review of all records at discharge with specific information on infectious source diagnosed and documented in the records by the physicians during hospital-stay. Focus of the infection were documented by bacterial culturing of possibly infected tissues and body fluids.

Information on death and readmission within the follow-up period were obtained from The Regional Zealand Patient Registration System, which is linked to the Danish Civil Registration System, with individual-level information on vital status of all Danish citizens [11].

### Statistical analyses

The primary endpoint was acute medical unplanned readmission (time to readmission) within the first 90 days of discharge after the index hospitalization.

Continuous data are presented as medians with interquartile ranges (IQR) assuming non-normality. We have compared groups by using differences within medians with 95% confidence intervals (CI) and exact differences of proportions with 95% CI for the difference. Differences were assumed significant if the 95% CI for the median difference or the 95% CI for the difference of proportions did not include zero.

We followed patients from the date of discharge from the index admission until the end of the follow-up period or until the time of readmission to hospital, emigration or death, whichever came first.

To measure the effects of covariates on the cumulative incidence of readmission we used competing-risk regression (Fine-Gray regression) to estimate unadjusted and adjusted subhazard ratios (aSHRs) with 95% confidence intervals (CI) for the covariates. The covariates (malignant disease, history of sepsis, CRP, Glucose, treatment with vasopressor, anticoagulants, diuretics and opioids) included in the initial full model were determined by statistical significance differences (95% CI on difference did not include zero) between readmitted and non-readmitted patients. However, selected variables (age, gender, chronic pulmonary disease, chronic kidney disease, diabetes) without significant differences according to readmission status in the crude analyses were also included in the regression models. Individual variables were adjusted for all other variables in the full regression model. In the final regression model, we have chosen a variable selection method with removing the weakest predictor from the list of covariates in the full model one by one until only significant variables remained in the final model. The statistic minus twice the logarithm of the maximized likelihood, −2logL, was used to compare different models fitted to the observed readmission data. The different models were compared by examining the change in value of -2logL on deleting a variable from a model or adding a variable to the model. Stratified regression analyses of diuretic treatment and malignant diseases as covariates were also performed after exclusion of patients with a history of heart failure or malignant disease with metastatic tumors according to the CCI classification.

Statistical analyses were performed using STATA v.15.1.

## Results

### Study population

**Fig. 1:**
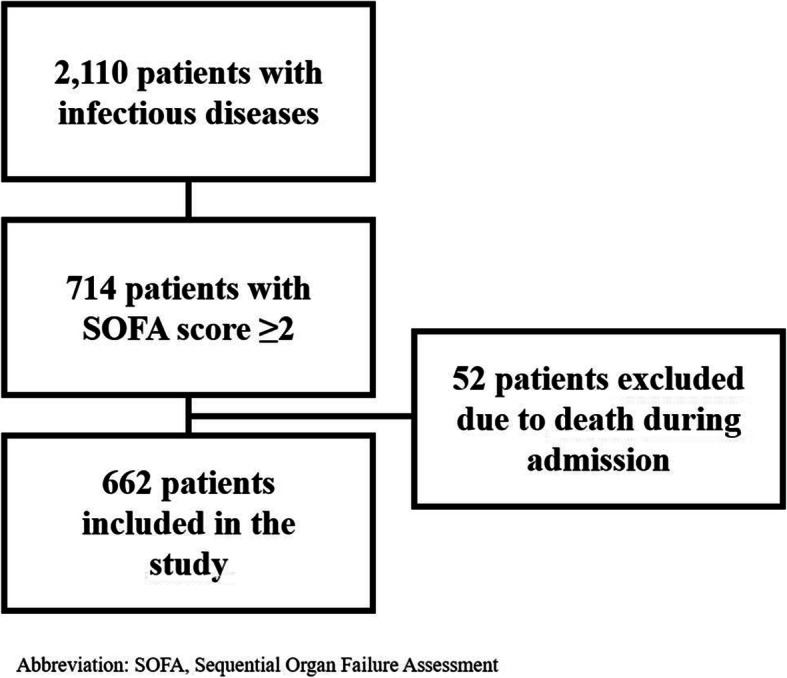
Study flow chart

A total of 12,092 patients were admitted to the ED during the study period and 3176 (26.3%) were treated with AB at arrival or within 24 h from arrival. A total of 1064 (33.5%) of the AB treated patients were excluded in the original study [5], and further two patients were excluded due to registration errors in the present study, leaving 2110 patients with infectious diseases (Fig. 1). A total of 714 (33.8%) patients had an increase of the SOFA score of at least two. A total of 52 patients had died during admission and were excluded from the present study leaving 662 patients, 44.1% female, with a median age of 74.8 (66.0–84.2) years for further analyses (Fig. 1).

### Readmission and baseline characteristics

A total of 237 (35.8, 95% CI 32.1–39.6%) were readmitted within 90 days after discharge and with a median time to readmission of 21 days (IQR 10–43 days).

Tables 1 and 2 shows baseline characteristics according to 90-day readmission. Readmitted patients had a higher comorbidity burden and had more frequently a history of admission for sepsis within the last year before the index admission (Table 1). Malignant diseases were more common among readmitted patients (Table 1). A total of 78 (45.1%) of the patients in the group CCI 3+ had a history of malignant diseases. Readmitted patients were more often treated with anticoagulants, diuretics and opioids at index admission (Table 2). There were no differences regarding age, gender, severity of sepsis based on total SOFA score and vital parameters (RR, blood oxygen saturation, SBP, HR, and Tp) on admission, length of stay (LOS), admission to ICU, white blood cell count, creatinine, bilirubin, platelets, lactate, the occurrence of atrial fibrillation, findings in blood cultures and source of infection (Table 1). The proportion of patients treated with vasopressor during the stay in the ICU and median values of CRP and glucose were higher in patients who were not readmitted (Table 1).

**Table 1:** Baseline characteristics according to 90-day readmission in septic patients

	Readmitted *N* = 237 (35.8%)	Not readmitted *N* = 425 (64.2%)	Difference (95% CI)
Female gender; n(%)^a^	105 (44.3)	187 (44.0)	0.3(÷7.5–8.2)
Age; median years (IQR)^a^	74.9 (64.7–83.9)	74.8 (66.6–84.4)	0.1(÷1.9–2.3)
Medical history			
Charlson Comorbidity Score; n(%)			
0	46 (19.4)	125 (29.4)	10.0 (3.4–16.6)
1–2	108 (45.6)	210 (49.4)	3.8(÷11.7–4.1)
3 + ^b^	83 (35.0)	90 (21.2)	13.8 (6.6–21.0)
Heart failure^a^	39 (16.5)	63 (14.8)	1.7(÷4.1–7.5)
Myocardial infarction/Ischemic heart disease^a^	33 (13.9)	53 (12.5)	1.4(÷4.0–6.8)
Chronic obstructive pulmonary disease^a^	64 (27.0)	96 (22.6)	4.4(÷2.5–11.3)
Malignant disease^b,c^	47 (19.8)	47 (11.0)	8.8 (2.9–14.9)
Diabetes^a,d^	55 (23.2)	85 (20.0)	3.2(÷3.4–9.8)
Kidney disease^a,e^	24 (10.1)	33 (7.8)	2.3(÷2.3–6.9)
Liver disease^a,f^	3 (1.3)	10 (2.4)	1.1(÷1.1–3.3)
Cerebrovascular disease^a,g^	49 (20.7)	75 (17.7)	3.0(÷3.3–9.3)
Hypertension^a^	89 (37.6)	154 (36.2)	1.4(÷6.2–9.1)
History of sepsis within last year before admission; n(%)^b^	87 (36.7)	106 (24.9)	11.8 (4.4–19.2)
Admission			
Length of stay; days (IQR)^a^	7 (4–10)	6 (3–9)	1(÷1–0)
Atrial fibrillation			
None, n(%)^a^	175 (73.8)	323 (76.0)	2.2(÷9.1–4.7)
New-onset atrial fibrillation; n(%)^a^	9 (3.8)	23 (5.4)	1.6(÷4.8–1.7)
History of atrial fibrillation; n(%)^a^	53 (22.4)	79 (18.6)	3.8(÷2.7–10.3)
Severity of disease			
Total SOFA score (IQR)^a^	3 (2–4)	3 (2–3)	0
Systolic blood pressure; mmHg, median (IQR)^a,h^	122 (105–144)	126 (109–147)	3(÷1–8)
Systolic blood pressure < 90 mmHg, n(%)^a^	13 (5.6)	20 (4.8)	0.8(÷2.8–4.4)
Respiratory rate; min^−1^, median (IQR)^a^	20 (17–24)	20 (18–24)	0
Heart rate; min^−1^, median (IQR)^a^	90 (80–105)	93 (77–109)	2(÷2–5)
O_2_-Saturation; %, median (IQR)^a^	95 (92–97)	95 (93–97)	0
Core temperature; ^o^Celcius, median (IQR)^a,i^	37.2 (36.6–38.0)	37.4 (36.7–38.4)	0.2 (0–0.4)
Altered mental state; n (%)^a^	62 (26.2)	117 (27.5)	1.3(÷8.3–5.7)
qSOFA score ≥ 2 on admission; n(%)^a^	48 (20.3)	78 (18.4)	1.9(÷4.4–8.2)
SIRS criteria ≥2 on admission, n(%)^a^	131 (55.3)	263 (61.9)	6.6(÷1.2–14.4)
Laboratory results			
CRP; median (IQR)^b^	65 (23–123)	82 (24–160)	10.9 (1.0–21.9)
White blood cell count ×10^9^/L, median (IQR)^a^	12.1 (8.9–16.1)	11.6 (8.6–16.6)	0.1(÷1.0–0.8)
Creatinine, μmol/L, median (IQR)^a^	115 (80–178)	113 (75–168)	5.0(÷15.0–4.0)
Bilirubin, mmol/L, median (IQR)^a^	10 (7–16)	10 (7–18)	1 (0–2)
Platelets, ×10^9^/L, median (IQR)^a^	216 (153–288)	205 (144–283)	10(÷26–5)
Lactate; mmol/L, median (IQR)^a,j^	1.2 (0.8–2.0)	1.3 (0.9–2.1)	0.1(÷0.1–0.2)
Glucose, mmol/L, median (IQR)^b^	6.8 (5.9–8.2)	7.3 (6.0–8.9)	0.4 (0.1–0.7)
Admission to intensive care unit, n (%)^a^	24 (10.1)	56 (13.2)	3.1(÷8.1–1.9)
Vasopressor, n (%)^b^	1 (0.4)	10 (2.4)	2.0 (0.3–3.7)
Mechanical ventilation, n (%)^a^	8 (3.4)	23 (5.4)	2.0(÷1.2–5.2)
Dialysis, n (%)^a^	1 (0.4)	2 (0.5)	0.1(÷1.1–0.9)
Positive blood cultures, n (%)^a,k^	18 (12.6)	38 (14.1)	1.5(÷6.8–3.9)
Source of infection			
Pulmonary, n (%)^a^	137 (57.8)	252 (59.3)	1.5(÷9.3–6.3)
Urine, n (%)^a^	47 (19.8)	101 (23.8)	4.0(÷10.5–2.5)
Abdominal, n (%)^a^	26 (11.0)	40 (9.4)	1.6(÷3.3–6.5)
Skin, n(%)^a^	19 (8.0)	26 (6.1)	1.9(÷2.2–6.0)
Endocarditis, n(%)^a^	1 (0.4)	1 (0.2)	0.2(÷0.7–1.1)
Central nervous system, n (%)^a^	1 (0.4)	3 (0.7)	0.3(÷1.4–0.8)
Facial/teeth, n(%)^a^	1 (0.4)	1 (0.2)	1.9(÷2.2–6.0)
Devices/implants	0	0	–
Others, n(%)^a^	3 (1.3)	0	1.3(÷0.1–2.7)
Unknown, n(%)^a^	19 (8.0)	35 (8.2)	0.2(÷4.5–4.1)
> 1 focus, n(%)^a^	18 (7.6)	33 (7.8)	0.2(÷4.4–4.0)

*CI* confidence interval, *CRP* C-reactive protein, *IQR* interquartile range, *qSOFA* quick Sequential Organ Failure Assessment, *SIRS* Systemic Inflammatory Response Syndrome, *SOFA* Sequential Organ Failure Assessment

^a^Ninety-five percent CI for the median difference or 95% CI for the difference of proportions included zero

^b^Ninety-five percent CI for the median difference or 95% CI for the difference of proportions did not include zero

^c^Tumor without metastasis, leukemia, lymphoma or metastatic tumors

^d^Diabetes with or without end organ damage

^e^Mild or severe kidney disease

^f^Mild or severe liver disease

^g^History of cerebrovascular disease including transient ischemic attack or a condition with hemiplegia

^h^Nine patients with missing blood pressure

^i^Fifteen patients with missing temperature

^j^(131/237 and 245/425) with measurement of lactate

^k^A total of 413 patients with blood cultures obtained

**Table 2:** Medical treatment on admission according to 90-day readmission in septic patients

Medication, N(%)	Readmitted *N* = 237 (35.8%)	Not readmitted *N* = 425 (64.2%)	Difference (95% CI)
Cardiac			
Beta-blockers^a^	75 (31.7)	112 (26.4)	5.3(÷2.0–12.6)
Calcium antagonists^a^	42 (17.7)	90 (21.2)	3.5(÷9.7–2.7)
Digoxin^a^	14 (5.9)	15 (3.5)	2.4(÷1.1–5.9)
ACE-inhibitors/AII-antagonists^a^	82 (34.6)	137 (32.2)	2.4(÷5.1–9.9)
Anticoagulants^b,c^	126 (53.2)	190 (44.7)	8.5 (0.6–16.4)
Diuretics^b^	126 (53.2)	167 (39.3)	13.9 (6.0–21.8)
Lipid lowering drugs^a^	79 (33.3)	119 (28.0)	5.3(÷2.0–12.7)
Pulmonary			
Inhalation medications^a^	58 (24.5)	89 (20.9)	3.6(÷3.1–10.3)
Diabetes			
Insulin/peroral antidiabetics^a^	42 (17.7)	74 (17.4)	0.3(÷5.7–6.3)
Psychopharmacology medication^a,d^	42 (17.7)	75 (17.7)	0
Analgesic			
Opioids^b^	51 (21.5)	62 (14.6)	6.9 (0.7–13.1)
Other analgesics^a,e^	78 (32.9)	147 (34.4)	1.5(÷9.0–6.0)

*ACE* Angiotensin-converting enzyme (ACE) inhibitors, *AII* angiotensin II receptor antagonists, *CI* confidence interval

^a^Ninety-five percent CI for the difference of proportions included zero

^b^Ninety-five percent CI for the difference of proportions did not include zero

^c^Acetylsalicylic acid, Non-Vitamin K antagonist oral anticoagulants, Warfarine/Dicoumarol

^d^Antipsychotics, Lithium, antidepressants, benzodiazepines

^e^Paracetamol, caffeine-phenazon, codeine, non-steroidal anti-inflammatory drugs, others

### Mortality

A total of 114 (17.2, 95% CI 14.4–20.3%) had died within 90 days after discharge and 54 (8.2%; 95% CI 6.2–10.5%) had died without being readmitted.

### Predictors for readmission

Unadjusted and adjusted SHR’s for the covariates are shown in Table 3. Malignant disease (aSHR 1.61, 95% CI 1.16–2.23), if previously admitted with sepsis within the last year before index admission (aSHR 1.41, 95% CI 1.08–1.84), and patients treated with diuretics on admission to the ED (aSHR 1.51, 95% CI 1.17–1.94) were independent predictors for readmission. aSHR (1.49, 1.13–1.96) for diuretic treatment was almost unchanged after exclusion of patients with heart failure, while aSHR (1.47; 0.96–2.25) for malignant disease was slightly attenuated after exclusion of patients with metastatic tumors.

**Table 3:** Unadjusted and adjusted competing-risks regression analyses of covariates and 90-day readmission among septic patients

	Unadjusted subhazard ratio	Adjusted subhazard ratio (full model)^a^ (95% CI)	Adjusted subhazard ratio (final model) (95% CI)
Age^b^	1.00 (0.99–1.01)	0.99 (0.98–1.00)	–
Female gender	1.02 (0.79–1.32)	1.06 (0.81–1.38)	–
COPD	1.24 (0.93–1.66)	1.16 (0.88–1.55)	–
Diabetes	1.12 (0.84–1.51)	1.09 (0.77–1.52)	–
Kidney disease	1.27 (0.83–1.94)	1.13 (0.74–1.70)	–
C-reactive protein^b^	0.998 (0.997–1.000)	1.00 (1.00–1.00)	–
Glucose^b^	0.99 (0.95–1.02)	0.98 (0.94–1.02)	–
Anticoagulant treatment	1.31 (1.01–1.68)	1.21 (0.91–1.61)	–
Vasopressor treatment	0.22 (0.03–1.72)	0.19 (0.02–1.58)	–
Opioid treatment	1.43 (1.05–1.94)	1.22 (0.90–1.67)	–
Malignant disease	1.70 (1.24–2.34)	1.68 (1.20–2.33)	1.61 (1.16–2.23)
Previous admitted with sepsis	1.51 (1.17–1.97)	1.28 (0.97–1.67)	1.41 (1.08–1.84)
Treatment with diuretics	1.53 (1.18–1.98)	1.48 (1.12–1.95)	1.51 (1.17–1.94)

*CI* confidence interval, *COPD* chronic obstructive pulmonary disease

^a^Variables adjusted for all other variables

^b^Increase of hazard ratio for one unit increase of the variable

In a new regression model where CCI was included, and after exclusion of patients with a history of cancer (*n* = 94), we found aSHR 1.16 (95% CI 0.82–1.65) and 1.41 (95% CI 0.91–2.20) (CCI 0 as reference) for patients with CCI 1–2 and CCI3+, respectively.

## Discussion

This study is the first to examine readmission within 90 days after sepsis based on prospective collected data among ED patients and using SOFA criteria for sepsis. We found that more than one-third of the patients were readmitted, and a history of malignant disease, a history with hospitalization for sepsis within the last year before the index admission, and treatment with diuretics were independent predictors for readmission. Variables reflecting the severity of sepsis did not differ between admitted and readmitted patients.

Different methods have previously been used to study readmission. Our study has included consecutively admitted patients with different severity of sepsis and discharged from either the ED, medical wards, or the ICU. Most of the previous research on readmission after sepsis are based on retrospective observational data in different settings and using other criteria (mainly SIRS) for sepsis than SOFA or qSOFA [7, 8, 12–24]. Some studies have only included severe sepsis or septic shock patients [8, 15, 20], or ICU patients [23]. Readmission rates after 30 or 90 days are typical outcome measures, 30 days being the most commonly used in previous research [7, 16, 17, 19, 21–24]. Many studies are based on hospital registry data [18, 19, 21, 23], or on claims data [7, 15, 16, 19, 20, 24]. Taken together, it is difficult to compare the study results because of the methodological differences.

### Readmission rate in other studies

Previous sepsis studies have reported 30-day readmissions rates ranging from 17.5 to 32.0% [8, 15, 17–19, 21–24]. Studies on 90-day readmissions showed readmission rates ranging from 30.7 to 42.6% [17–20].

### Predictors for readmission

We found diuretic treatment to be an independent predictor for readmission. This is not described in previous studies of sepsis. However, diuretic treatment as a predictor for readmission and hospitalization in general has previously been described [25–29]. Diuretics are commonly prescribed for patients with heart failure and treatment with diuretics has been found to be a strong predictor for readmission among patients with heart failure [28]. However, we found no significant difference in proportion of heart failure patients according to readmission status, and the risk of readmission among patients treated with diuretics was unchanged in an analysis of patients without heart failure. Another study [29] on sodium and potassium disorders in patients with community-acquired pneumonia found that use of diuretics was an independent predictor for 30-day readmission, although this study has no data on whether these patients had underlying heart failure. We cannot conclude from our data whether diuretic treatment has a causal effect on readmission, and if specific types of diuretics are associated with higher risk of readmission compared to others. Adverse reactions from drugs such as dehydration and electrolyte disturbances as potential causes of readmission should be explored in future studies. Further on, the predictive performance of diuretic treatment and the validity of diuretic treatment as an independent predictor variable needs to be examined in new prediction models in other sepsis cohorts before applying our results clinically.

We found that a history of malignant disease was an independent predictor of readmission which is supported by other studies [8, 18] on readmission of patients with sepsis. Our data did not allow us to conclude on the cause of acute readmission among patients with malignant diseases and if the readmissions were avoidable or unavoidable. We have no data on the disease activity and number of patients with advanced and terminal cancer. However, we have repeated the regression analyses among patients without metastatic tumors according to the CCI classification. aSHR for malignant disease in this group of patients was insignificantly increased which could be interpreted as patients with malignant diseases admitted with sepsis may have an increased risk of readmission despite the stage of disease.

Our study revealed that readmitted patients had an increased burden of comorbidities. However, almost half of the group of patients with high comorbidity burden (CCI 3+) were patients with malignant diseases and increased risk of readmission. Although the risk estimate was imprecise, the results of the stratified analysis of patients without malignancies can be interpreted as high comorbidity burden (CCI 3+) also increased the risk of readmission which is in accordance with other studies [15, 17, 19, 22, 23].

Our findings that prior admission for sepsis was associated with an increased risk of readmission is in agreement with previous research where prior hospitalizations were associated with 30-day readmission after sepsis [18, 19]. Previous sepsis admission may be a marker of the occurrence of other unmeasured factors that disposes to development of sepsis and also to increased risk of readmission in our study.

Unfortunately, we have no data on antimicrobial resistance (AMR). The surviving Sepsis Campaign guidelines [30] recommend empirical broad-spectrum antibiotic therapy for patients with sepsis and septic shock. It is well-known that this treatment may be harmful and carry a risk of AMR and may have other adverse consequences [31]. Whether AMR increase the risk of readmission through antibiotic-related adverse effects cannot be concluded from out study. However, AMR and other adverse effects to AB treatment should be considered in future studies.

Several predictors for readmission have been identified in previous studies: age [8, 17, 18], male gender [8], black race [8, 17], lower income [17], urban residence [17], non-elective index admission type [18], sources of payment for hospital stay [7, 8, 15], number of procedures during the index admission [18], ICU admission [19], severity of illness [15, 19], acute kidney failure [23], comorbid disease burden [15, 17, 19, 22, 23], diabetes [8, 22], chronic kidney disease [8, 22], chronic liver disease [8], chronic lung disease [8], congestive heart failure [22], collagen vascular disease [8], specific infectious agents [23], care facility after discharge [8, 21, 22], length of stay [8, 15, 19, 22], annual sepsis case volume [8], hospital sepsis mortality rate [8, 24], teaching hospital status [24], hospital quality metrics [24], low hemoglobin [18, 21], and high red cell distribution width [18].

Not all these factors were examined in our study. Age was not an independent risk factor for readmission in our study. We cannot report details for medical, social, or institutional support of older patients after discharge. However, discharge of older patients to nursing care facilities with potential mitigating effect on risk of readmission may have contributed to our findings.

Severity of sepsis including ICU admission, and LOS, was not associated with increased risk of readmission in our study. Baseline parameters and indicators of disease severity with good prognostic performance in relation to mortality such as SOFA are apparently not good prognosticators for readmission among septic patients. Previous studies have shown conflicting results regarding the relationship between illness severity and risk of readmission. Readmission risk was independent of illness severity and ICU admission status in one retrospective registry-based study [18], while severity of illness was associated with increased risk of readmission in two other registry-based retrospective studies [15, 19].

### Implications

The results can be helpful in developing readmission procedures and projects aiming at reducing the risk of readmission among septic patients. We suggest that the predictors identified in our study should be validated together with other relevant factors as predictor variables in future prediction models. However, we may suggest that patients with a history of malignant disease and increased comorbidity burden, previous admission for sepsis, and patients treated with diuretics, should be identified prior to discharge to ensure optimal management and follow-up of these patients.

### Strengths and limitations

Our study has several strengths. By use of a prospective study design we have identified all emergency admissions due to infections during the study period and we have used national registries with complete follow-up for readmission and mortality thereby reducing the risk of selection bias. Our cohort is population-based and includes patients from a uniform tax-supported health care system, which reduces the risk of referral bias. The identification of the sepsis cohort was based on the new Sepsis-3 criteria from 2016 [4].

The study has some limitations. First, the method used for calculation of the SOFA scores was not defined in the research protocol [5]. We have calculated the SOFA scores retrospectively and the method used to calculate SOFA in patients with chronic disorders with impact on the SOFA calculations has not been validated. Therefore, we cannot exclude the risk of misclassification of septic patients. Second, our study was not designed to identify several other relevant factors with potential impact on readmission such as socio-economical status [7] functional impairment [8], social support [14] and source control failure [32]. Prediction models that makes individual risk estimations of readmission should include such factors. Third, the numbers of included patients were low. A larger sample size could have made more precise estimates. Finally, this is a single center study which may limit the generalizability of our findings.

## Conclusion

More than one third of patients admitted with sepsis, and discharged alive, were readmitted within 90 days. A history of malignant disease, if previously admitted with sepsis, and diuretic treatment were independent predictors for 90-day readmission. Although the estimate was imprecise the risk of readmission was also increased in non-malignant patients with a CCI score of at least three.

## Data Availability

The datasets used are available by reasonable request to the corresponding author.

## Abbreviations

AB: Antibiotics
aSHR: Adjusted subhazard ratio
AF: Atrial fibrillation
AMR: Antimicrobial resistance
AVPU: Alert Voice Pain Unresponsive
CCI: Charlson comorbidity index
CI: Confidence interval
CRP: C-reactive protein
ECG: Electrocardiogram
ED: Emergency department
GCS: Glasgow coma scale
HR: Hazard ratio
ICU: Intensive care unit
IQR: Interquartile range
LOS: Length of stay
qSOFA: Quick sequential organ failure assessment
RR: Respiratory rate
SBP: Systolic blood pressure
SHR: Subhazard ratio
SIRS: Systemic inflammatory response syndrome
SOFA: Sequential organ faillure assessment
Tp: Temperature
